# Domiciliary Cough Monitoring for the Prediction of COPD Exacerbations

**DOI:** 10.1007/s00408-021-00435-9

**Published:** 2021-04-07

**Authors:** Michael G. Crooks, Albertus C. den Brinker, Susannah Thackray-Nocera, Ralph van Dinther, Caroline E. Wright, Alyn H. Morice

**Affiliations:** 1 Respiratory Medicine, Institute for Clinical and Applied Health Research, Hull York Medical School, Hull, UK; 2 Philips Research, Eindhoven, The Netherlands; 3 Respiratory Medicine, Hull University Teaching Hospitals NHS Trust, Cottingham, HU16 5JQ UK

**Keywords:** Chronic obstructive pulmonary disease, COPD, Exacerbation, Cough, Telehealth

## Abstract

**Introduction:**

Acute exacerbations of COPD (AE-COPD) are a leading cause of health service utilisation and are associated with morbidity and mortality. Identifying the prodrome of AE-COPD by monitoring symptoms and physiological parameters (telemonitoring) has proven disappointing and false alerts limit clinical utility. We report objective monitoring of cough counts around AE-COPD and the performance of a novel alert system identifying meaningful change in cough frequency.

**Methods:**

This prospective longitudinal study of cough monitoring included chronic obstructive pulmonary disease (COPD) patients experienced in telemonitoring that had two or more AE-COPD in the past year. Participants underwent cough monitoring and completed a daily questionnaire for 90 days. The automated system identified deteriorating trends in cough and this was compared with alerts generated by an established telemonitoring questionnaire.

**Results:**

28 patients [median age 66 (range 46–86), mean FEV-1% predicted 36% (SD 18%)] completed the study and had a total of 58 exacerbations (43 moderate and 15 severe). Alerts based on cough monitoring were generated mean 3.4 days before 45% of AE-COPD with one false alert every 100 days. In contrast, questionnaire-based alerts occurred in the prodrome of 88% of AE-COPD with one false alert every 10 days.

**Conclusion:**

An alert system based on cough frequency alone predicted 45% AE-COPD; the low false alert rate with cough monitoring suggests it is a practical and clinically relevant tool. In contrast, the utility of questionnaire-based symptom monitoring is limited by frequent false alerts.

## Introduction

Chronic obstructive pulmonary disease (COPD) is the third leading cause of death worldwide [1]. AE-COPD negatively impact patients’ quality of life and are a leading cause of health service utilisation, adding to the global challenge [2]. Attempts have been made to identify impending AE-COPD with the objective of early intervention to improve outcomes. Remote monitoring of patients’ symptoms and physiological parameters (telemonitoring) may be used for this purpose with ‘alerts’ conveyed to a clinician when worsening symptoms/signs are detected.

However, there is no compelling evidence that existing telemonitoring strategies can detect meaningful change in clinical condition. Randomised controlled trials of telemonitoring in COPD have failed to demonstrate a reduction in hospitalisations [3–6]. Indeed, an increase in the number of community treated exacerbations and increased health service utilisation have been reported in patients receiving telehealth [3, 6]. One problem with existing telemonitoring approaches is the frequency of ‘false alerts’ i.e. those not representing an impending exacerbation that will require treatment. In a well-conducted COPD telemonitoring trial, community teams dealt with 2441 alerts from 97 participants over a 1-year period (25 alerts per patient per year) [3] thus leading to high resource utilisation.

Cough and sputum production are common in COPD [7, 8] and are associated with exacerbations, lung function decline and risk of death [9]. Increased cough is common during AE-COPD and a prodrome of increasing symptoms including cough can be seen for up to 2 weeks before AE-COPD [10]. Calverley et al. demonstrated a mean increase in symptom score of around 1 unit (scale: 0–4) at the time of exacerbation across a range of symptoms including cough, breathlessness, chest tightness and night-time wakening. We have previously demonstrated a similar change in cough score measured using a 5-point Likert scale during AE-COPD convalescence [11]. Whilst change of this magnitude can be detected at a population level; in an individual patient it is unlikely to prove clinically useful. Cough is the symptom with the highest concordance with AE-COPD, independent of COPD severity [10]. It is therefore a most promising candidate for predicting AE-COPD.

We have previously described a system that analyses acoustic signal from microphones placed in the domestic environment to identify and record cough events [12]. This system can detect individual long-term cough count trends and demonstrate a reduction in cough frequency during AE-COPD convalescence [11]. In the current study, we monitored cough frequency in exacerbation prone COPD patients over 90 days and assessed the predictive performance of an automated AE-COPD alert system based on cough count and compare it with an established telehealth questionnaire.

## Methods

### Study Design

We conducted a prospective longitudinal study of continual cough monitoring in COPD patients experienced in telemonitoring. The study design has been described briefly elsewhere [13]; further detail is provided here. To provide a reasonable chance of detecting AE-COPD, participants were studied for 90 days using domiciliary cough monitoring and asked to complete daily questionnaires each morning. The association between cough frequency and AE-COPD was analysed to determine the greatest probability of exacerbation prediction whilst minimising false alerts.

The study was reviewed and approved by the North East-York Research Ethics Committee (REC Ref: 15/N/0291), the United Kingdom Health Research Authority and the Internal Committee Biomedical Experiments of Philips Research.

### Participants

Participants were identified by the local clinical team as participating in the community-based COPD telemonitoring program and having had two or more AE-COPD in the past year. Participants were excluded if they failed to provide consent or they had a comorbid condition that was considered likely to affect their cough frequency or if they were unable to adhere to monitoring. All participants provided written informed consent.

### Cough Monitoring

#### Cough Monitoring System

The cough monitor comprised a stationary microphone (Jabra 510+, Jabra GN, Denmark) and a laptop computer (HP 630, Hewlett-Packard, California, USA) that were wirelessly paired. We selected the sleeping area in which to position the microphone (typically the bedside table) based on our previous observation that day and night time cough frequencies are highly correlated [12].

The cough monitoring system is unobtrusive and does not require user input after initial installation. Participants were visited monthly by a study team member to confirm that the monitor was functioning correctly and to download monitoring data. Participants were asked to contact the study team if the system failed between visits, indicated by a change in colour of an indicator light on the microphone. Cough monitoring data were analysed at the end of the study by an independent observer and trends in cough count identified.

#### Cough Count Analysis

The cough monitoring system continuously analyses audio input, only storing audio features compatible with cough. A limited number of associated 1-s audio excerpts were used as controls for validation and classifier training. This approach preserves the privacy of patients and cohabitees by ensuring that no intelligible speech is recorded. The stored audio excerpts allow personalisation of the cough classifier for optimal performance using semi-supervised learning [11, 13]. The machine learning algorithm used in this classifier is XGboost.

For the purpose of monitoring trends in cough count over time, specificity is preferred over sensitivity to ensure a high positive predictive value. The threshold for cough classification was set at 0.9 (i.e. if a cough is detected by the cough monitor, there is a 90% probability that the identified sound is a true cough). This paradigm reduces the number of false positives and will boost the positive predictive value given the high prevalence of non-cough events [14].

Cough counts were aggregated over a 24 h period running from noon to noon. These counts were mapped onto the B-scale [13]. The B-scale was introduced to harmonise the day-to-day variations across patients based on the finding that for stable patients, the standard deviation was nearly linearly dependent on the average cough count. Mapping of the cough count provides a convenient way to capture this phenomenon, where the variability in the mapped data is independent of its (patient-specific) mean. The mapping reads:

$$B = \alpha \log \left( {1 + \beta C} \right),$$

where *C* is the cough count, *B* is its mapped value and *α* and *β* are the mapping constants for night-time cough count totals given by *α* = 3.45 and *β* = 0.04 [13].

### Symptom Monitoring

An established questionnaire was used to record participants’ symptoms and COPD-related medication use daily [3]. The symptom questionnaire consists of eight weighted yes/no questions (total score: 0–11). An alert is raised if the score rises to 5 or more or to 4 for 2 consecutive days.

### Acute Exacerbations of COPD (AE-COPD)

Moderate and severe COPD exacerbations were identified retrospectively by one of the following:

- Patient self-reporting [answering ‘yes’ to steroid (prednisolone) and/or antibiotic use for their chest on the medication component of the daily questionnaire]
- Review of primary care prescriptions
- Hospital admission records

A moderate exacerbation was defined as requiring treatment with oral corticosteroids and/or antibiotics for two or more consecutive days without requiring hospital attendance. A severe COPD exacerbation was defined as an exacerbation requiring hospital attendance.

### Data Analysis

#### Establishing Cough Count Trend in the Period Around AE-COPD

The date of AE-COPD onset was defined as the first day that an individual answered ‘yes’ to taking steroids and/or antibiotic (self-report) or the date a prescription was generated (prescribing data). This date was regarded as day 0 and cough counts were analysed for 8 days before and after this day. To prevent recurrent exacerbations and missing data from interfering in the analysis, only epochs of complete cough monitoring data that did not overlap with other AE-COPD periods were included.

#### Relationship Between Questionnaire Reported Cough and Objective Cough Count

To investigate the relationship between self-reported increased cough and objective cough count we calculated the correlation coefficient for self-reported change in cough (binary Yes/No) and (i) the change in cough count compared with the day before and (ii) the cough count represented on the B-scale.

#### Evaluation of Questionnaire and Cough Monitor Alert Performance in Predicting AE-COPD

The alert system was developed using data from this longitudinal study and therefore this represents the derivation cohort. Alert system development is described in detail elsewhere [13]. Briefly, individual baseline cough frequency was established and deviation from this assessed using the B-scale. An alert was generated if the cough frequency exceeded the pre-specified threshold on at least 2 out of 3 consecutive days.

To assess alert performance we analysed success rate, false alarm rate, and lead time.

##### Success Rate

If one or more alerts were raised in the 8 days before an AE-COPD, this was considered a successful identification of an upcoming AE-COPD. AE-COPD periods less than 8 days apart were treated as a single event on the basis that inadequate time had elapsed to allow recovery. The success rate of the alert system was defined as the number of identified upcoming AE-COPD relative to the total number of AE-COPD.

##### False Alarm Rate

The false alarm rate was determined by identifying the number of alerts generated on days that were not associated with an AE-COPD relative to the total number of days not associated with AE-COPD. Days not associated with an AE-COPD are all monitoring days except those from start to end of an AE-COPD and 8 days preceding start and 8 days after end.

##### Lead Time

The lead time is calculated as the number of days between an alert and the start of the AE-COPD (Day 0). In cases of multiple alerts preceding the AE-COPD, the earliest warning was taken. This metric indicates the duration of advanced warning of an upcoming AE-COPD.

## Results

### Subject Characteristics

**Fig. 1:**
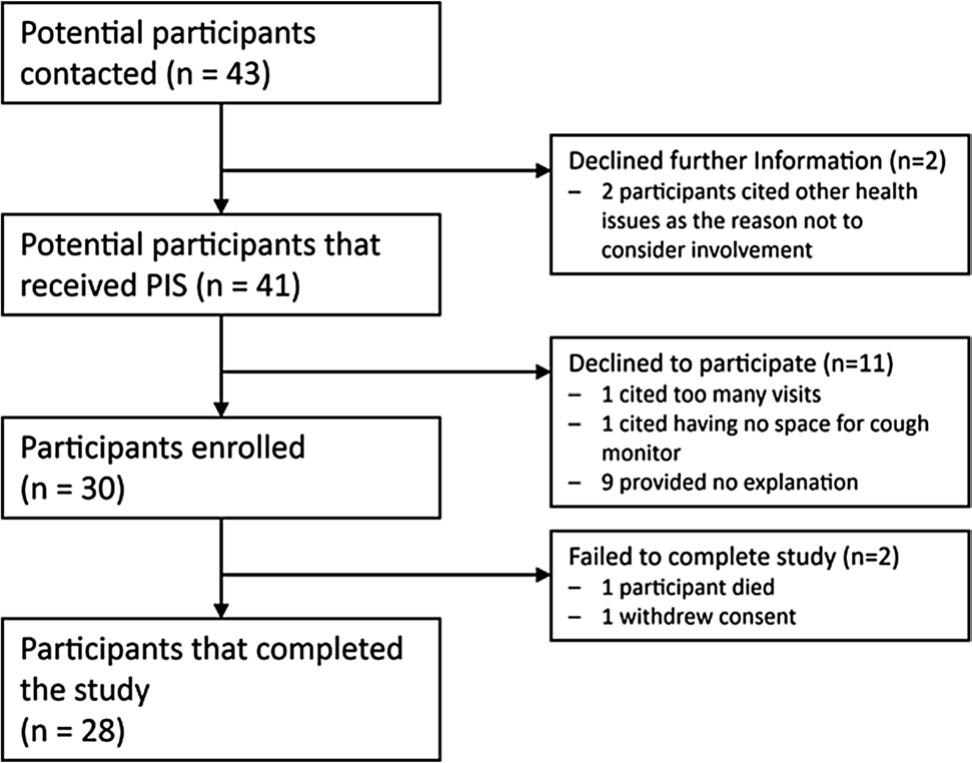
Consort diagram demonstrating participant recruitment and retention

A consort diagram illustrating recruitment, participation and retention is presented in Fig. 1. Participants were recruited over a 14 month period spanning all seasons. Of the 28 participants that completed the study, two were discovered to have a malfunctioning cough monitor making the collected data uninterpretable and a third participant was excluded from analysis because this patient consistently reported a near maximum increase in symptoms (suggesting inappropriate questionnaire responses). 27 participants were included in analysis of daily questionnaire monitoring and 25 participants for cough monitoring. Study participants’ characteristics are presented in Table 1.

**Table 1:** Baseline demographics for study participants

Characteristic	All (*n* = 28)	Male (*n* = 16)	Female (*n* = 12)
Age (years)	66 (46–86)	65 (46–77)	67 (53–86)
BMI	25 (10)	26 (12)	24 (5)
Smoking status			
Current/ex/unknown	6/21/1	3/12/1	3/9/0
Pack years	35 (0–240)	35 (0–240)	37 (3–70)
FEV-1 (L)	0.87 (0.51)	0.98 (0.6)	0.70 (0.27)
% predicted FEV-1	36 (18)	38 (21)	34 (12)
CAT score	28 (8)	29 (6)	27 (10)

Values are expressed as mean (SD) or median (range) depending on distribution

*BMI* body mass index, *FEV-1* forced expiratory volume in 1 s, *CAT* COPD assessment test

#### Cough Count Trend in the Period Around AE-COPD

A total of 58 exacerbations (43 moderate and 15 severe) occurred in the 28 patients completing the study. 22 patients experienced at least one exacerbation.

**Fig. 2:**
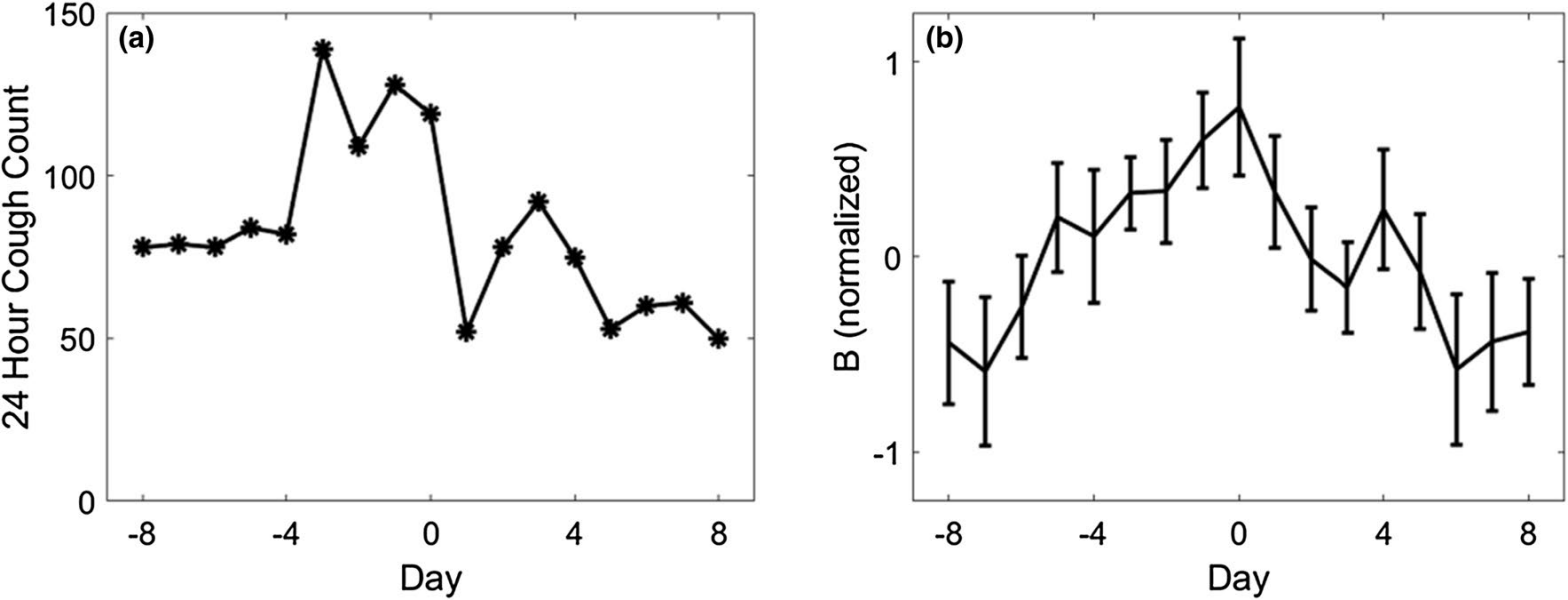
**a** An individual participant’s total 24-h cough counts in the period around AE-COPD. **b** Mean Cough Count around the time of all included AE-COPD presented on the B-scale. The data are normalised so that the average cough count is 0 on the B-scale for all patients. Error bars represent the standard error of the mean

Cough monitoring data were available for 25 patients who had a total of 53 AE-COPD (38 moderate and 15 severe). 15 moderate and one severe AE-COPD had complete monitoring data for the full 17 day exacerbation period and did not overlap with other AE-COPD periods and therefore met requirements for this analysis. Cough count trends around the time of AE-COPD are presented in Fig. 2.

#### Relationship Between Questionnaire Reported Cough and Objective Cough Count

Combined questionnaire and cough count data were available for 24 participants. From a total of 1840 questionnaire responses, 878 indicated an increased cough on that day.

There was no correlation between participants’ response to the question ‘Do you have an increased cough today’ and change in measured cough count over the previous 24 h (correlation coefficient 0.06 SD 0.1). A weak correlation was observed for reported increase in cough and measured cough count displayed on the B-scale (correlation coefficient 0.24 SD 0.23).

#### Alert Performance Based on Daily Symptom Questionnaire or Cough Monitoring to Identify Impending AE-COPD

The performance of alerts based on Daily Symptom Questionnaire Scores and Cough Monitoring are presented in Table 2. 88% of AE-COPD had a preceding alert based on the daily symptom questionnaire compared with 45% based on cough monitoring. However, Daily Symptom Questionnaire monitoring generated around one false alert every 10 days compared with one false alert every 100 days based on cough monitoring.

**Table 2:** Performance of alerts based on questionnaire or cough monitoring data

	Sensitivity			False alerts		
	Total number AE-COPD	AE-COPD with preceding alert	Success rate	Total number of days with no associated AE-COPD	Total number of alerts on days with no associated AE-COPD	False alert rate
Questionnaire-based alerts^a^	42	37	0.88	1084	109	0.101
Cough monitor-based alerts^b^	31	14	0.45	1062	13	0.012

Success rate was determined as the number of identified AE-COPD relative to the total number of AE-COPD. The false alarm rate was determined as the number of alerts generated on days that were not associated with an AE-COPD relative to the total number of days not associated with AE-COPD

^a^*n* = 27 had adequate data to assess performance of questionnaire-based alerts

^b^*n* = 22 had adequate data to assess performance of cough monitor-based alerts

The lead time i.e. the time between alerts based on daily symptom questionnaires or cough monitoring and the corresponding AE-COPD were 3.4 (SD 2.9) days and 3.4 (SD 2.8) days respectively.

## Discussion

We report the first longitudinal study of continual daily cough monitoring in COPD patients specifically designed to assess cough frequency in the AE-COPD prodrome. Observational studies have shown that patients report worsening cough for several days before starting treatment [10]. For the first time, we confirm an increase in objectively measured cough frequency occurs using a cough monitoring system that analyses ambient sound with a static microphone located in the persons sleeping quarters.

The cough monitoring alert system detected increased cough frequency a mean of 3.4 days before approximately half of AE-COPD. This was achieved with fewer false alerts than a paradigm based on questionnaires. The majority of questionnaire-based alerts in our predictive window of 8-days prior to an AE-COPD can be explained as alerts occurring by chance. Indeed the questionnaire-based system generated one false alert every 10 days, so the chance of an alert occurring in the window prior to an AE-COPD can often be explained by random association. Conversely, there is a high probability that an alert generated by cough monitoring signifies a genuine change in clinical state associated with impending AE-COPD. We detected 14 alerts pre-exacerbation with only 13 false alerts during the study. The false alert rate was one per 100 days. The average lead time of 3.4 days suggests that cough monitoring-based alerts could prompt a meaningful early clinical response.

Cough monitoring in this study was limited to the room where participants slept. It was considered that there may be inter and intra-participant variation in the amount of time spent in this room. However, analysing data based on patient proximity to the microphone or during set night time hours did not alter the overall classifier performance [13].

Cough monitoring-based alerts are individualised by establishing each patient’s baseline cough count and identifying deviation from it. The daily symptom questionnaire attempts to do the same by asking patients to report a change from their normal state. However, it was clear that some patients continually answered ‘yes’ to questions such as ‘Are you more breathless than usual today?’. In order to identify if establishing an individualised baseline was the reason for favourable cough monitoring-based alert performance, we subsequently analysed the daily symptom questionnaire data using the methodology used for cough monitor alert generation i.e. looking for an increase in symptom score over an individualised baseline. The questionnaire success rate dropped to 0.41 and the false alarm rate to 0.015. This performance remained numerically inferior to the cough monitoring system, but most notable was the reduction in lead time to an average of 1 day giving little predictive value.

Participants in this study have severe COPD with a high symptom burden and experienced frequent exacerbations. This provided an opportunity to observe cough frequency during AE-COPD but made establishing a baseline during clinical stability challenging. Some participants were excluded from analysis because they did not have adequate time between AE-COPD to allow assessment of a baseline. The studied population is a challenging cohort due to their clinical instability. It is possible that performance will improve further in individuals with lower exacerbation frequency.

In this study, we define AE-COPD according to treatment and healthcare utilisation. This means that mild AE-COPD, defined as symptom worsening for more than two consecutive days but not leading to treatment with systemic steroids or antibiotics [15], are not included in our analysis. Although these events are often reported in studies where symptom diaries are completed, their importance in the context of clinical interventions generated through telemonitoring is not clear. One could argue that questionnaire-based alerts that did not precede a moderate or severe AE-COPD were not false-alerts, but alerted clinicians to a mild AE-COPD. However, these events resolved without the need for additional interventions or health care utilisation. For the purpose of telemonitoring, a system that differentiates mild AE-COPD that will resolve spontaneously from impending moderate-severe AE-COPD that may benefit from early treatment provides optimal healthcare resource use.

In addition to the favourable performance of cough monitoring-based exacerbation prediction; auditory cough monitoring is a passive process that requires no direct user input. A drawback of conventional telemonitoring is the reliance on patients to correctly interpret and respond to questionnaires and input correctly recorded physiological measurements. We have previously reported poor adherence with home spirometry monitoring [12] and in this study, we observed a number of participants that repetitively reported their symptoms as having ‘increased’ in severity, limiting the utility of questionnaire-based reporting.

In previous studies, we have observed reluctance from patients and their carers to engage with a system which continuously monitors all sounds within the home environment [12]. There are a number of ethical and data protection issues around this method of observation. Early concerns about the acceptability of having audio recording equipment within the home appear to have been adequately addressed by limiting recorded sound to 1 s excerpts; ensuring no possibility of conversational speech being recorded.

This study has a number of limitations. Firstly, it was a derivation cohort used to develop the alert system based on the results of objective cough monitoring. System performance will need validation in a different cohort of patients; ideally, a heterogeneous group of COPD patients to determine the generalisability of the method. The static domiciliary system used may well be applicable to a group of severe, mainly house-bound individuals and further work will be required to determine whether performance is maintained in less sedentary patients. The impact of cough among other family members may be a greater factor when a less severe cohort of patients is studied. Although we very successfully reduced the number of false alerts (increasing specificity), the sensitivity of the system may be less than optimal. Finally, the hardware used in this study was ‘off-the-shelf’ and as such was prone to remedial technical failure in two out of the 30 systems deployed. Reliability may be improved by bespoke technology.

## Conclusion

An increased cough count can be detected in the prodrome of around half AE-COPD using a cough monitoring system that analyses ambient sound in patients’ sleeping quarters. This can be achieved with a low false alert rate, meaning that each alert can be considered meaningful by clinicians. This contrasts with questionnaire-based alerts, which are difficult to interpret due to the high false positive rate.

The cough monitoring-based alert system described in this study warrants further assessment in a validation cohort. If alert performance is confirmed, cough monitoring will offer the first objective, passive monitoring strategy for COPD patients that is capable of generating meaningful clinical alerts of impending AE-COPD.
